# Defining Global Health Diplomacy: Assessing United States Global Health Diplomacy actors’ knowledge, skills, and competencies

**DOI:** 10.1371/journal.pgph.0005422

**Published:** 2026-05-20

**Authors:** Floramae Esapebong-Ray, Karen McDonnell, Rebecca Katz

**Affiliations:** 1 Former Council on Foreign Relations International Affairs Fellow, Department of State, Bureau of Global Health Security and Diplomacy, Washington, District of Columbia, United States of America; 2 Department of Prevention and Community Health, Milken Institute School of Public Health, George Washington University, Washington, District of Columbia, United States of America; 3 Center for Global Health Science and Security, Georgetown University Medical Center, Washington, District of Columbia, United States of America; University of Essex, UNITED KINGDOM OF GREAT BRITAIN AND NORTHERN IRELAND

## Abstract

The COVID-19 pandemic fundamentally reshaped the field of Global Health Diplomacy (GHD), necessitating a coordinated international response that strived for equitable access to limited health resources. The pandemic, however, also exposed gaps in GHD actors’ preparedness and response capabilities, which impacted their ability to navigate critical global health challenges. Addressing gaps in the knowledge, skills, and competencies of GHD actors is crucial to ensuring a more effective response to emerging infectious diseases and health challenges, particularly amid reduced foreign assistance. This study, conducted between December 1, 2023, and January 10, 2024, explores the knowledge, skills, and competencies required by U.S. GHD actors using an integrated Grounded Theory and descriptive research design. It identifies thematic similarities and differences across Core, Multistakeholder, and Informal GHD actors, offering actionable recommendations for tailored GHD training and competency-building. The findings provide critical insights into the professionalization of GHD and its role in advancing global health security and diplomacy in a post-pandemic era. With ongoing resource constraints, geopolitical shifts, declining multilateralism, and changes to the field of global health, strengthening GHD is even more vital for addressing emerging health threats and building future resilience.

## Introduction

The international response to the COVID-19 pandemic involved bilateral and multilateral diplomatic engagements, often negotiated by an emerging cadre of global health diplomats. These health diplomats worked to strengthen health systems, enhance global health security and preparedness, and increase equitable access to COVID-19 vaccines, diagnostics, and treatments. They often faced significant economic and resource barriers, challenges related to misinformation and disinformation, and ongoing geopolitical tensions that complicated the pandemic response [1], while balancing international assistance with growing domestic health system demands.

Despite these issues, the COVID-19 pandemic elevated health as a foreign policy priority and underscored the significance of Global Health Diplomacy (GHD), highlighting the relevance of collaboration, communication, coordination, and cooperation in addressing health threats of this magnitude [2]. Across the globe, Ministries of Foreign Affairs recognized the need for qualified diplomats to tackle global health security challenges [3]. On March 14, 2024, the U.S. Department of State announced the establishment of a Foreign Ministry Channel (FMC) to convene Ministers of Foreign Affairs and strengthen the capacity of diplomatic staff to respond to global health security threats [4]. With ongoing resource constraints, reduced foreign assistance, declining multilateralism, and changes to the global health landscape, strengthening GHD is even more vital for addressing emerging health threats and building future resilience [5]. The shift from donor reliance to increased local ownership is critical as it transforms global health engagement from dependency-driven aid to strategic partnerships that promote shared interests, co-investment, mutual accountability, and national sustainability [6].

This study sought to identify the knowledge, skills, and competencies required by self-identified global health diplomats working on behalf of the United States Government during the COVID-19 response between January 2020 and January 2024, for effective GHD practice. We acknowledge the ongoing debate about what constitutes successful Global Health Diplomacy and to whom, as well as the subjectivity of what defines ‘effectiveness’ and ‘practice’. Therefore, our research approach allowed us to examine the essential GHD knowledge, skills, and competencies from the perspective of GHD actors, including the barriers and challenges they encountered during the pandemic. We relied on participants’ self-identified values, perceptions, and lived experiences, which were categorized into knowledge, skills, and competencies.

We operationalized the study concepts as follows: Skills refer to specific learned abilities required to practice Global Health Diplomacy. Knowledge (technical knowledge) refers to the practical or theoretical understanding of Global Health Diplomacy that informs GHD practice. Competencies are the integrated combination of essential skills, knowledge, and abilities that GHD actors need to perform effectively in real-world GHD settings. Core Competencies refer to the timely, context-appropriate application of competencies (knowledge, skills, and abilities) in real-world GHD practice to achieve intended outcomes. Core competencies answer the question: Can GHD actors apply technical knowledge, skills, and abilities effectively at the right place and time to produce the desired impact? Global Health Diplomacy practice refers to the real-world application and implementation of GHD core competencies to address global health challenges and achieve global health-related goals. In summary, technical knowledge and skills form the foundation, competencies represent the integration of these abilities, and core competencies reflect effective, context-specific performance in GHD practice.

Our research questions were as follows:

1. What skills do GHD actors need for Global Health Diplomacy practice?
2. What competencies do GHD actors need for Global Health Diplomacy practice?
3. What technical knowledge gaps exist amongst Global Health Diplomacy actors?
4. How do Global Health Diplomacy actors define Global Health Diplomacy in a post-COVID era?

Much has changed in the U.S. Government over the past year, including the practice of Global Health Diplomacy. One such change is a notable shift from the term Global Health Diplomacy to Health Diplomacy, reflecting a more decentralized, diversified, and integrated approach that incorporates a wider range of non-state actors. This study, conducted in 2023–2024, captures the experiences of U.S. career health diplomats and other GHD actors prior to the major changes brought about by the Trump administration in 2025.

## Methods

### Ethics statement

This study was conducted in accordance with Institutional Review Board (IRB) approval [FWA00005945] granted on November 14, 2023, by the George Washington University Ethics Review Board. The study was determined to be IRB-exempt as it posed no more than minimal risk to study participants. All participants received informed consent forms via email before their inclusion in the study, and verbal consent was obtained prior to commencing the interview, as per IRB approval.

### Research design

Our study employed an integrated Grounded Theory and descriptive research design, by adopting a pragmatic approach that prioritized contributing to change and quality improvement in GHD practice over furthering conceptual understanding [7]. The descriptive research methodology focused on presenting phenomena, including viewpoints, trends, beliefs, and perceptions of the study population. Purposeful criterion and snowball sampling were used to select self-identified U.S. GHD actors from a pool of Core, Multistakeholder, and Informal GHD actors, defined by the taxonomy of GHD actors proposed by Katz et al., 2011 [8], and expanded on by Brown et al., 2014 [9] (see Table 1).

**Table 1:** Description of global health diplomacy actors.

GHD Actor Category	Description
**Core Global Health Diplomacy Actors**	Formal members of the diplomatic corps, specialized diplomats, and Health Attachés with principal responsibilities of fostering interactions between governments, reporting, negotiating, and developing agreements between and among states and governments.
**Multistakeholder Global Health Diplomacy Actors**	Government employees and representatives of multilateral organizations, responsible for negotiating and facilitating interactions and partnerships between and among state, non-state, and multilateral actors.
**Informal Global Health Diplomacy Actors**	Representatives from the private sector, non-governmental organizations, civil society organizations, academia, government employees, and the public. They are responsible for interactions between public health actors and field counterparts and support pandemic prevention, preparedness, and response by informing program and policy design and implementation.

We established self-identification of U.S. GHD actors through a structured recruitment and screening process to identify and verify participants’ direct engagement in GHD practice. This included explicit self-description, standardized screening questions, individual titles/roles, and experience criteria to ensure they had the knowledge, expertise, or background to address the research questions. Where available, we also verified this information using participants’ professional profiles (LinkedIn or X) and biographies on official websites. We purposefully recruited individuals whose work (titles, roles, and responsibilities) was associated with U.S. government–related GHD functions as described by Table 1, even if they were stationed in other countries. We also required at least two years of relevant GHD employment, specifically during the COVID-19 pandemic, and those who spoke English as a first language or were fluent in English. All but one participant had a combination of U.S.-based and international GHD experience.

During the recruitment process and at the start of interviews, we asked prospective participants to describe their professional roles and responsibilities. To confirm their engagement in GHD, we also asked them the following screening questions, which were directly adapted from the description of GHD actors in Table 1:

1. Does your role involve fostering interactions between governments, reporting, negotiating, and/or developing agreements among states and governments?
2. Have you participated in processes that integrate health priorities into foreign policy, development cooperation, or multilateral agreements?
3. Does your role involve health negotiations and the facilitation of interactions and partnerships among state, non-state, and multilateral actors?
4. Does your role involve interactions between public health actors and field counterparts to support pandemic prevention, preparedness, and response by informing program and policy design and implementation?

Affirmative responses to one or more of the four screening questions were required for inclusion. Following recruitment, two members of the research team independently classified prospective participants into Core, Multistakeholder, and Informal categories according to the Taxonomy of GHD actors, based on their responses to screening questions, functional GHD roles and responsibilities, and institutional affiliations. We then compared our classification and resolved any discrepancies through discussion to enhance reliability. GHD actor taxonomic classification also helped ensure representation across all three actor categories during participant recruitment.

We conducted semi-structured, in-depth interviews using a pilot-tested guide to ensure a structured approach. Using open-ended questions, we asked participants to reflect on their personal experiences before, during, and after the pandemic, including their overall perspective on the GHD landscape and potential implications for GHD leadership, policy, and practice. We collected data between December 1, 2023, and January 10, 2024, via the Zoom platform. We contacted 71 people via email and LinkedIn; 32 responded, 11 declined or deferred participation due to scheduling conflicts, and 21 completed interviews: Core (7), Multistakeholder (7), and Informal (7). Interview lengths ranged between 35 and 60 minutes. Three transcripts, one from each GHD actor category, were used to establish intercoder reliability, which informed coding of the remaining transcripts. Open coding was conducted using the constant comparative method in Dedoose qualitative data analysis software, and responses were categorized into GHD skills, competencies, knowledge, and definitions [10]. We used Braun & Clarke’s six steps for systematic thematic analysis to identify recurring information patterns generated through the coding process, which we organized into themes and sub-themes [11].

## Results

Our results present a thematic analysis of 21 interviews with self-identified practicing global health diplomats associated with the U.S. Government who were engaged in the COVID-19 response. Although the findings are based on participants’ subjective accounts, these actors share a commonality: the COVID-19 pandemic was an unprecedented defining moment during which they practiced Global Health Diplomacy while balancing various roles and responsibilities. We identified six key themes in respondents’ assessments of the knowledge, skills, and competencies required for Global Health Diplomacy. These include interpersonal, technical, critical and analytical thinking, practical/practice-based, learning, and communication.

For each of these themes, participants’ responses have been distilled and summarized into core competencies below.

### Global health diplomacy core competencies

Earlier, we defined core competencies as the essential knowledge, skills, and abilities that GHD actors need to practice Global Health Diplomacy, and which must be applied at the right place and time.

#### Interpersonal core competencies.

Interpersonal core competencies are traits, skills, and abilities that GHD actors say are essential for harmonious interactions with others, including those from different cultural backgrounds, to help build and maintain trusted and genuine diplomatic relationships.

Respondents emphasized the importance of personal attributes such as empathy, humility, patience, tolerance for ambiguity, flexibility, emotional intelligence, and active listening in all diplomatic interactions. Cultural awareness and humility were recognized as essential for understanding nuances, nonverbal cues, and fostering trust and cooperation. GHD actors stated that these core competencies are crucial for understanding stakeholders’ priorities, perspectives, interests, and motivations during negotiations, particularly when navigating cultural differences. Sincerity, honesty, open-mindedness, and trustworthiness were also stressed as critical for building rapport, managing challenging bilateral relationships, and establishing credibility. Respondents noted that these qualities facilitate networking, the formation of sustainable partnerships, and collaboration, which are essential for leveraging existing systems and resources to advance public and global health objectives.

Key technical knowledge gaps included a lack of collaboration and teamwork, particularly in bilateral and multilateral interactions. These gaps sometimes presented as countries’ reluctance or inability to share data, biospecimens, and resources which hindered a more coordinated and effective pandemic response.

#### Technical core competencies.

Technical core competencies refer to specialized technical expertise that GHD actors can leverage while practicing Global Health Diplomacy. Clinical or public health expertise, financial stewardship, project and program management, and data analysis were deemed essential for GHD.

Core GHD actors emphasized that technical expertise is crucial for establishing credibility and trust. Some participants within this group asserted the importance of having a clinical or health background to better understand disease surveillance, clinical trials, and diagnostics. Conversely, others argued that being a generalist with a broad range of health and non-health expertise was equally valuable, as it enabled Core GHD actors to leverage others’ expertise. Respondents in this group also stressed the significance of logistics and supply chain management, biostatistics, scientific communication, and the ability to navigate the cultures of the U.S. government and non-governmental organizations (NGOs).

Multistakeholder GHD actors emphasized the importance of being perceived as subject matter experts to build legitimacy during negotiations with foreign counterparts. Key competencies included project monitoring and evaluation, creating after-action reports, managing and utilizing data, conducting research, computer skills, technical negotiations, and sharing data and resources during emergency responses. These GHD respondents also highlighted the importance of emergency preparedness and response, and the value of last-mile public health laboratory systems in detecting outbreaks. In contrast, informal GHD actors pointed to One Health and systems-thinking as essential for addressing emerging health threats.

When discussing gaps in technical knowledge, Core and Informal GHD actors mentioned a lack of understanding of global health regulations, instruments, and treaties, informatics and web technology, and country performance capacity assessments and frameworks. Core GHD actors identified gaps in understanding legal frameworks that specifically address liabilities related to administering new drugs or vaccines during emergencies. They also mentioned supply chain management, clinical and public health leadership, and understanding of foreign policy. Multistakeholder GHD actors noted gaps in knowledge of vaccines, diagnostics, therapeutics, epidemiology, and statistics. They stressed the importance of distinguishing between incidence and prevalence rates and of communicating these trends effectively to the public. Informal GHD actors identified gaps in knowledge of One Health, systems-thinking, emergency preparedness, and risk management.

#### Critical and analytical thinking core competencies.

Critical and analytical thinking core competencies refer to the ability of GHD actors to synthesize, analyze, and evaluate information objectively and logically, to make informed and sound decisions.

Participants who were career diplomats or Foreign Service Officers with GHD in their portfolios recognized these core competencies as crucial for the U.S. State Department’s Foreign Service and specialists’ roles [12]. For all three GHD actor categories, countering misinformation and disinformation in real time across social and mainstream media outlets during an infodemic was a significant challenge, as was accessing and distinguishing credible information sources. Core GHD actors stressed the importance of balancing risk and uncertainty, particularly when dealing with limited data or insufficient understanding of the long-term consequences of medical countermeasures. Being able to find credible information, assess its quality, and make informed decisions amid incomplete evidence was a recurring theme within this group. However, key knowledge gaps remained, including a lack of understanding of the principles underlying diagnostic and preventive measures, the limitations of scientific data, and how to make informed trade-offs in the face of incomplete data.

Both Multistakeholder and Informal GHD actors emphasized the importance of situational and contextual understanding, including political, socioeconomic, legal, technological, and environmental factors. Multistakeholder GHD actors highlighted the need to reassess global health partnerships post-pandemic to facilitate equitable health outcomes. Conversely, Informal GHD actors emphasized the importance of evaluating the successes and failures of the COVID-19 response to inform future GHD training initiatives and strengthen health systems.

### Practical or practice-based core competencies

Practical or practice-based core competencies collectively refer to GHD actors’ expression of demonstrable knowledge, skills, and abilities employed in real-world GHD settings, which extend beyond a theoretical understanding of Global Health Diplomacy. Given the variation of respondents’ roles and responsibilities within and across GHD actor categories, these core competencies are not one-size-fits-all and reflect this diversity.

Core GHD actors highlighted the importance of traditional diplomatic skills in professional interactions and communication, especially when managing multiple perspectives or representing opposing views. Negotiation and mediation are crucial for navigating power imbalances in donor-recipient relationships and setting priorities. Participants within this group emphasized the importance of understanding how governments communicate and utilize diplomatic channels for formal démarches or complaints. They also pointed out the importance of understanding U.S. interagency coordination protocols, global governance frameworks, and multisectoral stakeholder engagement. Finding a balance between being persuasive and prescriptive in bilateral or multilateral discussions was key, particularly when engaging with policymakers. Core GHD actors also noted the importance of understanding the intersection of health, foreign policy priorities, the economy, and meta crises, such as forced migration, conflict, and political or ethnic tensions, and the reasons why global health programs often fail. While recognizing the importance of formal GHD training for a more effective crisis response, they recommended incorporating discussion-based case studies to examine real-world GHD events and scenarios to support the development of critical thinking and improve decision-making. Simulation exercises, mentorship from seasoned health diplomats, and peer-to-peer learning were also deemed important for GHD practice.

Multistakeholder GHD actors emphasized the importance of combining practical experience across health and non-health sectors with training in GHD and health security to tackle global health challenges. They highlighted adaptability, flexibility, risk-taking, crisis leadership, and negotiation as essential for GHD practice. Respondents within this group also noted the importance of real-world GHD experience, stakeholder engagement, and situational awareness in managing power dynamics within donor-recipient relationships. They recommended reevaluating personal and institutional biases and empowering partners to assert their agency during negotiations. They deemed training in GHD, emergency preparedness, global health security, public health, and interpersonal competencies essential.

Informal GHD actors stressed the importance of real-world experience in pandemic preparedness and response, strengthening health systems, public health ethics, and engaging multisector stakeholders. They highlighted the need for interdisciplinary training in One Health, Essential Public Health Functions, health economics, and industrial psychology, and called for updates to medical and public health curricula to tackle 21st-century challenges. Respondents also emphasized the importance of understanding political, governmental, and non-governmental systems, stakeholder engagement, and geopolitical tensions between the global health community and foreign policymakers. One participant noted that GHD competencies are often influenced by U.S. foreign policy priorities, making it hard to define them in advance.

Practice-based technical knowledge gaps also varied among GHD actor groups. Core GHD actors highlighted a lack of real-world GHD experience, a need for a deeper understanding of the U.S. emergency response system, and for clearer roles and responsibilities among federal agencies. They also noted a lack of knowledge about financial accountability and about implementing lockdowns or stay-at-home orders without causing severe economic disruption. Multistakeholder GHD actors highlighted leadership challenges stemming from limited health-related technical expertise and an understanding of GHD. They also mentioned the misuse of diplomacy for disruptive or non-altruistic purposes.

Conversely, Informal GHD actors expressed limited understanding of the Public Health Ethics framework and its application at the individual, community, and population levels. Respondents in this group also highlighted gaps in knowledge of geopolitics, U.S. foreign policy, and foreign diplomats’ perspectives on the evolving global health landscape. Additional knowledge gaps included insufficient understanding of the U.S. emergency response system, stakeholder analyses, effective use of community resources, and recognition of how politics and political will influence health policies.

#### Learning core competencies.

Learning core competencies refers to the processes through which GHD actors acquire knowledge, information, skills, or abilities from past experiences that lead to positive change and improvement in GHD practice.

Respondents across all GHD actor categories underscored widespread misinformation and disinformation on social media platforms during COVID-19, which they said significantly influenced the public’s perception of both scientific and non-scientific discussions, and efforts to control the virus’s spread. They noted that science and public health were questioned, widely rejected, polarized, and discredited in unprecedented ways. Participants stressed that GHD actors needed to learn to manage and address misinformation and disinformation and understand their impact on the credibility of public health practitioners, policymakers, and GHD actors. They also identified social media literacy and leveraging lessons from past outbreaks to prepare for future pandemics as key knowledge gaps. Additionally, respondents highlighted the importance of continuous learning and on-the-job learning through systemic or multisectoral approaches to develop technical skills relevant to their roles and to adapt to changing responsibilities and unexpected situations.

Core GHD actors emphasized the importance of knowing how to rapidly acquire the required knowledge and skills, noting that they often rely on just-in-time information and trial-and-error methods to assess the reliability of sources. Knowing how to maintain diplomatic channels for the exchange of real-time information was crucial for making informed decisions, as was the consideration of health in all policies. Respondents emphasized the importance of understanding the private sector’s role in developing diagnostics and therapeutics, managing supply chains, and engaging global health diplomats in discussions on global security and international relations. Conversely, Multistakeholder GHD actors stressed the importance of understanding the roles and functions of the U.S. Government’s Legislative, Executive, and Judicial branches.

### Communication core competencies

Communication Core Competencies refer to the effective use of various communication formats by GHD actors, including written and verbal communication, public speaking, elevator pitches, social media, and crisis/risk communication in GHD practice. Respondents in all three GHD actor groups highlighted the use of multiple communication formats, including crisis/risk and emergency communication. They also emphasized the importance of stakeholder knowledge, multisectoral engagement, and how to leverage social media amid an infodemic.

Core GHD actors stated the importance of proficiency in various communication formats, synthesizing and translating technical information for non-technical audiences, public speaking, and presenting scientific evidence to decision-makers. They emphasized the role of foreign language skills in fostering cross-cultural understanding and navigating nuanced conversations. They also underscored the importance of effectively communicating risk and uncertainty to diverse audiences, particularly during emergencies, and understanding how mainstream and social media influence public health messaging and behavior.

Multistakeholder GHD actors said effective communication is a relational process that fosters trust and collaboration among partners, necessitating precise, authentic, and audience-specific language. They highlighted the importance of foreign language skills in enabling direct communication, building rapport, and understanding cultural nuances. The ability to deliver concise, accurate messaging tailored to the audience, particularly when conveying risk with limited or rapidly changing information, was essential.

Informal GHD actors emphasized the importance of communicating effectively with diverse stakeholders, delivering clear, concise presentations, and rapidly disseminating information in emergencies. They view communication as a vital tool for negotiations, leadership, and diplomacy. Specific competencies include public speaking, conveying risk and uncertainty without causing alarm, leveraging community health workers, and engaging populations during health interventions.

While discussing technical knowledge gaps in communication, Core and Informal GHD actors identified challenges, including difficulty accessing information promptly, communicating risk as a continuum, and addressing the politicization of health and erosion of public trust. Multistakeholder GHD actors noted gaps in the sharing of scientific information, in foreign-language skills, and in effective communication during emergencies. Informal GHD actors highlighted difficulties in quickly accessing and sharing information, especially in lower-income countries and among last-mile populations, as well as challenges in translating scientific data for non-expert audiences.

**Fig 1:**
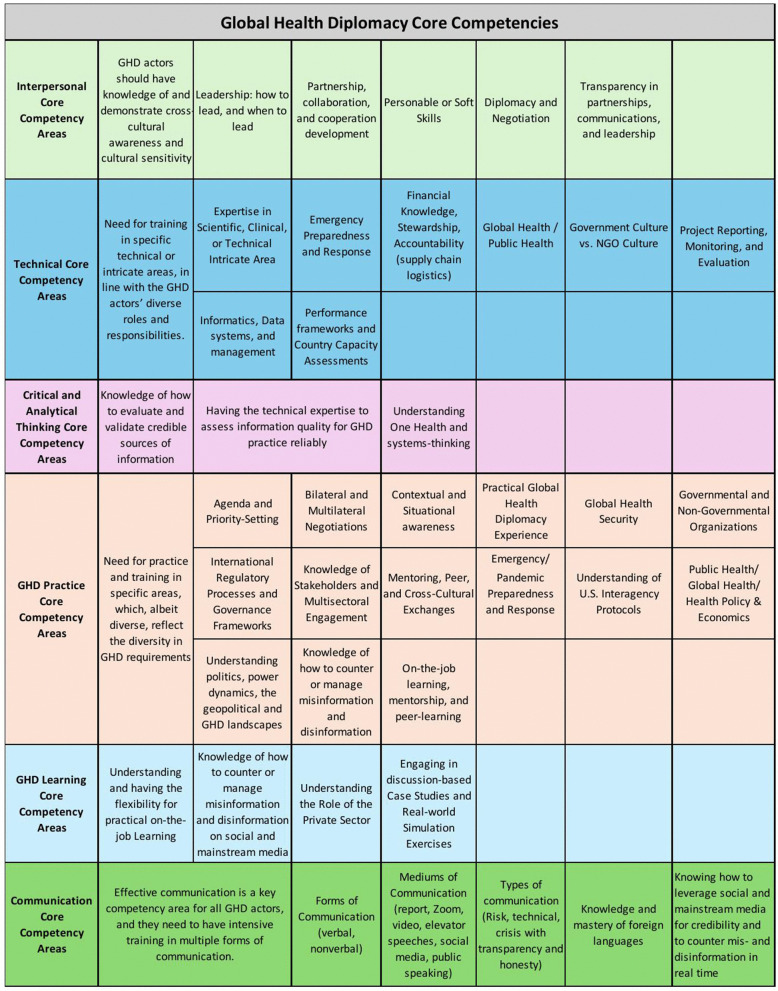
A summary of required global health diplomacy core competencies.

Fig 1 below presents a list of required Global Health Diplomacy core competencies, synthesized from responses by Core, Multistakeholder, and Informal GHD actors regarding required GHD knowledge, skills, and competencies. These core competencies were aggregated across all three GHD actor groups from respective Interpersonal, Technical, Critical and Analytical Thinking, Practical, Learning, and Communication themes and corresponding sub-themes.

We have provided an example using interpersonal core competencies to demonstrate how we derived the domains in Fig 1. These core competency areas are an aggregate of the interpersonal competency themes and corresponding sub-themes derived from the responses of Core, Multistakeholder, and Informal GHD actors to the required GHD knowledge, skills, and competencies. Aggregated interpersonal sub-themes were then synthesized into their most appropriate descriptors or labels, such as “Leadership: how to lead and when to lead”, or “partnership collaboration and cooperation development”. We contextualized descriptors or labels within the participants’ experiences and responses to enhance the validity of interpretation and applicability to real-world settings.

To avoid duplication, if a sub-theme appeared more than once, for instance, “Leadership: how to lead and when to lead” appeared as a sub-theme from both Multistakeholder and Informal GHD actors’ responses, it was only represented once in Fig 1. To enhance the reliability of this process, two research team members independently derived core competency descriptors or labels, which were then compared and reconciled to ensure they were clear, accurately represented the data, and to resolve any discrepancies. Complementary to Fig 1, Fig 3 presents a visual representation of sub-themes (knowledge, skills, and abilities) within and across GHD actor groups, illustrating both distinct and overlapping sub-themes.

### Defining global health diplomacy in a new era

We asked participants to define Global Health Diplomacy to assess how previous definitions may have addressed or overlooked the demands of the evolving global health diplomacy, geopolitical, and geoeconomic landscape.

Core GHD actors’ definitions acknowledge the profound impact of global health challenges and the interconnection between health and all sectors of society. They emphasize the importance of leveraging health for population advocacy, strengthening international relations, fostering opportunities for cooperation and collaboration, and advancing foreign policy priorities to achieve better, more equitable global health outcomes.

Multistakeholder GHD actors’ definitions emphasized the need for a multifaceted, evidence-based, and scientific approach to tackling global health challenges through One Health. Respondents also stated the importance of global multisectoral, multistakeholder communication, coordination, and collaboration that involve local communities and organizations in outbreak prevention and response efforts.

Informal GHD actors’ definitions emphasized a collaborative approach to improve global disease surveillance and preparedness for future outbreaks by strengthening countries’ capacities and health systems. They stated that GHD can enhance human well-being by bridging the gap between scientific discovery and application. Understanding the changing geopolitical landscape and recognizing the interconnectedness between humans, animals, and plants was crucial for addressing health challenges effectively.

## Discussion

This research directly addresses calls for the professionalization of Global Health Diplomacy by identifying the essential knowledge, skills, and competencies for effective GHD practice. In 2022, Brown advocated for the establishment of a U.S. Foreign Health Service, which further emphasized the need for structured GHD training and applied practice across all GHD actor groups [13].

While applying a cross-cutting practice framework acknowledges that GHD practice is inherently integrative, the data support treating practical/practice-based core competencies as a separate analytic domain. Across GHD actor groups, respondents consistently distinguish between having competencies and delivering results in practice. Their accounts emphasized the need to select, adapt, and integrate the other core competencies within specific real-world political, institutional, and operational constraints. They also identified personal challenges and situations in which the other core competencies or training were present but difficult to apply in practice, supporting the analytical distinction of practical/practice-based core competencies. These challenges and reported gaps reflect GHD actors’ performance in context, such as negotiating political complexities, navigating power asymmetries, or responding to outbreaks or crises, rather than the absence of knowledge, skills, or abilities.

This separate-domain framing also recognizes differences in the roles and responsibilities of GHD actors and the uniqueness of GHD interactions. It emphasizes role-specific, context-dependent performance that involves situational judgment, strategic timing, and adaptation in GHD practice, rather than uniform behavior or standards. For instance, some participants emphasized the distinction between possessing the required competencies and successfully applying them in real-world settings, such as pandemic agreement negotiations, shaping foreign policy agendas, or advancing public health goals with bilateral counterparts, while others did not. Participants’ accounts show that a cross-cutting framing would understate the qualitative differences that participants perceived and expressed. Therefore, we established practical/practice-based core competencies as a separate domain to capture GHD actors’ ability to effectively translate knowledge, skills, and abilities into action to achieve desired outcomes in diverse real-world GHD settings.

Similarly, specific training recommendations, including simulations, mentorship, and case-based learning, target GHD practice as distinct developmental competency domains, tailored to actors’ needs. It also allows for performance evaluation of specific practice-based domains across various GHD actor roles. These findings suggest that distinction is crucial in a field where effectiveness and impact depend on real-world execution rather than theoretical proficiency alone.

Effective GHD practice requires actors to appropriately apply a combination of core competencies (knowledge, skills, and abilities) at the right place and time, underscoring the multidisciplinary nature of GHD and the diverse roles and responsibilities of GHD actors. The results indicate that equipping GHD actors will be even more critical in a resource-constrained era that requires them to navigate the complexities of poly- and meta crises, while safeguarding health.

Thematic similarities and differences observed among GHD actor categories highlight areas of convergence and divergence rather than unanimity in their responses. For example, key core competencies, such as effective communication and stakeholder knowledge and engagement, were emphasized across all GHD actor groups. These may reflect the centrality of communication in navigating dynamic settings and the importance of multisector stakeholder involvement in GHD. Variations in technical and practice-based core competencies suggest that GHD training must be tailored to address actors’ specific needs and competency gaps. This proposition aligns with calls for a clear professionalization pathway for Global Health Diplomacy and for tailored approaches to competency-building.

Global health diplomats operate across political and economic divides, cultures, and values, often balancing national interests with global needs. Participants’ accounts revealed that building relationships and finding common ground in foreign policy is especially crucial when working with health attachés, foreign affairs ministers, ambassadors, and ministers of health. Being personable fosters open dialogue, helping GHD actors manage complex diplomatic interactions and develop strategic partnerships. These core competencies are vital for tackling complex global challenges, fostering collaboration, and improving efficiency in emergency response efforts [14]. Understanding counterparts’ perspectives and positions in multilateral forums such as the World Health Assembly and the United Nations General Assembly [15] can promote trust, collaboration, and cooperation. Self-reflection and understanding of political, institutional, and organizational cultures are also essential when implementing programs in countries with different sociopolitical contexts. Similarly, cross-cultural awareness and cultural sensitivity [16] can enhance communication by increasing receptivity, acceptability, and diplomatic tact during exchanges.

Global Health Diplomacy discussions are increasingly focused on funding sources, their use, the importance of evidence-based, data-driven negotiations, and accountability to the U.S. Legislative branch, American taxpayers, and institutional donors. Flexibility in resource allocation is essential for rapid deployment and adapting resources to meet the needs of lower-income and last-mile populations during emergencies. Knowing when and how to lead during these interactions enables strategic influence without overreach, especially in fragile alliances. Although leadership and transparency were especially critical during the last pandemic, they were often challenged by mis-and disinformation, politicization, polarization, and a decline in the credibility of political leaders and the scientific community. Therefore, building trust and credibility is key to maintaining long-term legitimacy, especially when navigating the complexities of public health recommendations and scientific evidence during an infodemic.

Having a mix of clinical, public health, and biomedical expertise supports a deeper understanding of epidemiology, disease transmission, medical countermeasures, vaccine trials, and core public health functions, such as prevention, protection, and promotion [17]. Similarly, a mastery of data analytics, informatics, and performance frameworks can help legitimize GHD actors’ advocacy efforts. Conversely, as generalists, GHD actors often possess additional cross-cutting competencies from prior experience to drive policy development, decision-making, and action [18], even with limited formal training. Nevertheless, tailored training is essential to address specific technical knowledge gaps to equip actors to navigate complex systems, influence policy, respond effectively to crises, and promote equitable and sustainable health solutions [19].

Critical and analytical thinking, along with the application of systems-thinking approaches, such as One Health, facilitate the development of holistic strategies to address health complexities and their broader implications for trade, travel, and global economies [20,21]. Examining the interconnectedness between climate change, disease outbreaks, food security, and migration is essential for designing, implementing, and coordinating multi-level, multisectoral public health programs to support integrated solutions. Similarly, logic and objectivity in decision-making rely on the ability to evaluate, synthesize, and apply information effectively, particularly when human lives are at stake. Adeptly applying the Public Health Ethics frameworks to interventions [22], such as vaccine prioritization/emergency use, vaccine or mask mandates, stay-at-home orders, and lockdowns, requires balancing risk and uncertainty. Much of this involves assessing potential impacts and outcomes not only on local populations but also on bilateral and multilateral relationships, and the economy.

Understanding the intersection of health and U.S. foreign policy is essential to represent U.S. interests and objectives adequately [23] and to ensure limited resources are directed toward high-impact, achievable goals. GHD actors need to recognize the role of agenda and priority-setting, political will, leadership transitions, geopolitical rivalries [24], and U.S. foreign policy influence on global health priorities within the current geoeconomic landscape. Analyzing political, socioeconomic, technological, environmental, and legal contexts that influence present and future global health partnerships [25] can facilitate more equitable agreements and sustainable impact. This process requires situational and contextual awareness to assess political and power dynamics in real time. It also helps GHD actors evaluate countries’ capacities to prevent, detect, and respond to disease outbreaks, and adopt systems-based approaches that target and address root causes rather than just symptoms.

Practical (first-hand or field-based) GHD experience is crucial, as theory alone cannot fully prepare GHD actors for the complexities and dynamics of Global Health Diplomacy. Stakeholder engagement is central to GHD and facilitates bilateral, multilateral, national, and local interactions [26]. It also facilitates negotiations and the understanding of target populations and living contexts, thereby guiding the development of tailored health policies and interventions. The increase in emerging threats, including biosecurity risks, pandemics, climate- and conflict-related diseases, and mass migration, warrants proactive preparedness and response. Strengthening public health systems through resource infusion, public health promotion and protection efforts can enhance their resilience to global shocks amid the notable decline in foreign assistance. Knowing how to access and use credible information in emergencies supports adaptability and informed decision-making [27], secures critical resources, manages uncertainty, allocates resources more efficiently, and provides timely responses [28]. Additionally, learning from past outbreaks [29,30] supports proactive measures and helps break the recurring cycle of panic and neglect in infectious disease responses [31].

Practical/real-world GHD experience through mentorship and peer exchanges further supports experiential learning, enabling GHD actors to develop and refine new skills through observation and application. Mentoring entry- and mid-level GHD actors may help build continuity of expertise, increase practitioner confidence, and strengthen intergenerational and cross-cultural resilience. Ongoing and on-the-job learning in rapidly changing contexts enables GHD actors to remain agile and adaptable. Simulation exercises and case studies based on real events can be used for training, to elicit critical and analytical thinking, improve decision-making skills, and assess strengths and weaknesses in emergency responses. Furthermore, understanding private-sector operations and leveraging their capabilities in emergencies complements and enhances public-sector efforts. As public funding and foreign assistance shrink, the role of the private sector will be even more crucial in enabling faster, more efficient responses to critical resource and systemic gaps, such as vaccines, diagnostics, personal protective equipment (PPE), and supply chains.

Communication in all formats, including written, spoken, presentations, and non-verbal, is critical, as it serves as the vehicle through which multisectoral coordination, collaboration [32], and diplomacy occur. While effective communication relies on relational skills, proficiency in foreign languages also facilitates negotiations, cross-cultural interactions, and understanding, thereby circumventing the need to filter information through translators and interpreters [33,34]. Managing misinformation and disinformation across social and mainstream media requires continuous, adaptive strategies to counter populist skepticism and politicization, which have severely undermined public trust [35] and disease-prevention efforts. By framing public health messaging appropriately in polarized societies, GHD actors can help foster public understanding and engagement and mitigate the erosion of credibility in leadership and policymakers. Furthermore, carefully designing emergency messaging and risk communication can improve clarity and accessibility while addressing barriers to individual and community adherence stemming from perceptions of susceptibility, severity, benefits, threats, and self-efficacy.

### Implications for global health diplomacy leadership, policy, and practice

Despite recognizing the interrelatedness of health and national security post-COVID, global financing for health priorities has declined amid rising infectious disease burdens, emerging threats, and mass migration. As multilateral cooperation declines and artificial intelligence (AI) and other modern technologies spread rapidly, our findings underscore that tackling global health issues may increasingly rely on a strategic form of diplomacy that offers flexibility in problem-solving. This approach involves forming adaptable, purpose-driven, overlapping partnerships and coalitions, which participants emphasized leverage specific expertise, capacity, resources, and strengths across countries, civil society, and the private sector, rather than relying solely on traditional alliances. Known as strategic geometric diplomacy or variable geometry diplomacy in U.S. foreign policy [36], it may also be applied to ecosystems, plurilateral, and regional challenges, including security, climate change, trade, and strategic competition [5,6]. This approach also embodies the U.S. government’s shift in terminology from Global Health Diplomacy to Health Diplomacy.

For U.S. health diplomats, these findings are particularly relevant, as they can support the development of tailored, evidence-based training and resources, which respondents emphasized will better equip them to navigate the challenges and demands of this new era. With the dissolution of USAID, diminished investment in multilateral organizations, deglobalization, and reduced U.S. foreign assistance, U.S.-funded global health programs are being redesigned through bilateral agreements to reinforce local ownership in preparation for transition and sustainability. Taken together, these findings suggest that U.S. diplomats will need to adeptly negotiate, advocate for, and implement policies and interventions that align with current U.S. foreign policy objectives and safeguard the public’s health.

**Fig 2:**
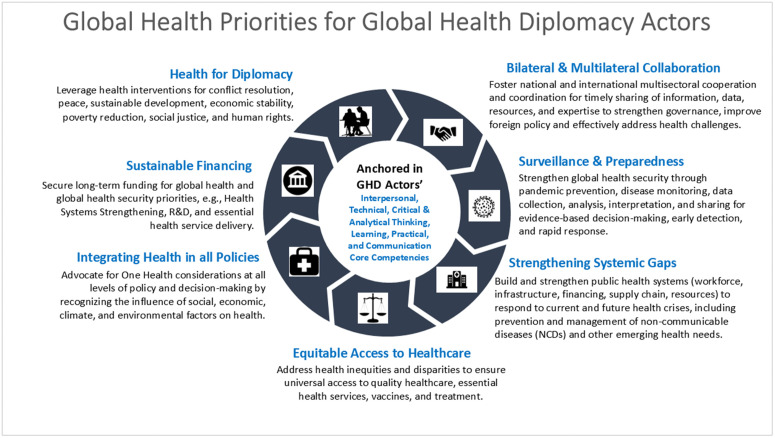
Conceptual framework of global health priorities for global health diplomacy actors.

Based on our results, we also propose a novel conceptual framework of seven key priority domains through which GHD actors can strengthen global health through leadership, policy, and practice. These domains are health for diplomacy; bilateral and multilateral collaboration; surveillance and preparedness; strengthening systemic gaps; equitable access to healthcare; integrating health into all policies; and sustainable funding. This framework (**Fig 2**) depicts and emphasizes the interconnectedness of these domains, underpinned by GHD actors’ practical application of their technical knowledge, skills, and abilities in interpersonal interactions, critical and analytical thinking, learning, and communication to real-life GHD events.

**Fig 3:**
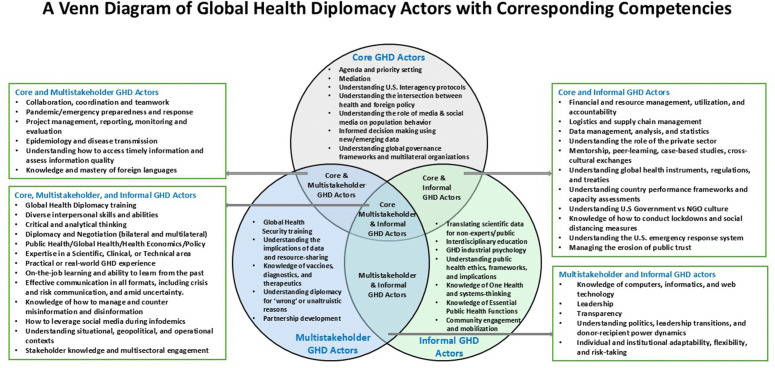
A venn diagram of global health diplomacy actors with corresponding competencies.

Katz et al. (2011) proposed a taxonomy classifying Global Health Diplomacy (GHD) actors into three distinct categories: Core, Multistakeholder, and Informal [8], which was previously depicted by a Global Health Diplomacy Pyramid by Brown et al. (2014) [9]. However, the results of this study suggest that a more accurate representation of present-day GHD actors may be achieved through a three-circle Venn diagram (Fig 3). The figure also illustrates the distribution of reported knowledge, skills, and abilities (derived from themes and sub-themes) within and across all three GHD actor groups. The overlapping sections represent shared competencies and collaborative opportunities, while distinct areas emphasize each GHD actor category’s unique contributions and challenges faced. This visual framework underscores the interconnectedness of GHD actors and the evolving nature of GHD amid shifting roles, responsibilities, foreign policy priorities, resource constraints, and geopolitics.

## Limitations and conclusions

Global Health Diplomacy is pivotal to addressing global health challenges, particularly in the era of pandemic-driven change, evolving geopolitics, and reductions in global health funding. Our study uniquely focuses on the knowledge, skills, and competencies required for GHD practice across all three actor categories, offering actionable insights to guide the professionalization and advancement of GHD in a rapidly evolving global context.

While centered on U.S. GHD actors, the findings draw on respondents’ domestic (U.S.-based) and global experiences and may apply to diverse international contexts, thereby bridging the gap between scholarship and practice. We recommend tailored approaches to building GHD core competencies and training, interdisciplinary collaboration, and implementing hybrid training models to achieve broader reach and address the varied proficiency levels of GHD actors amid shifting roles and responsibilities. For example, training programs may be adapted to mirror the National Institutes of Health (NIH) five-level proficiency scale for each core competency: Basic/entry-level (fundamental awareness), Novice (limited experience), Intermediate (mid-career), Proficient (senior-level), and Expert (recognized authority) [37].

As global health faces increasing resource constraints, GHD emerges as a cornerstone for navigating these challenges and strengthening collaborative approaches to address critical gaps that go beyond technocratic acumen. By fostering strategic partnerships, adeptly navigating the intersections of health and foreign policy, and addressing inequities, GHD actors can strengthen global health security, address emerging threats, and improve health outcomes. Further research is needed to examine GHD competencies in other geographical contexts, including non-democratic states, to develop a more comprehensive understanding of GHD practice amid funding reductions and the proliferation of artificial intelligence.
